# Supplementary material for: Family structure and the association with physical activity—Findings from 40 countries participating in the Health Behaviour in School-aged Children (HBSC) study
Source: PLoS One. 2024 Apr 17;19(4):e0300188. doi: 10.1371/journal.pone.0300188 (PMC11023480; doi:10.1371/journal.pone.0300188)
Supplement: Attachment — Submitted filename: Response to Reviewers.docx [file pone.0300188.s001.docx]

# Response letter to the Editor and reviewers 29.11.2023

First, we would like to thank the Editor for the opportunity to resubmit a revised version of our manuscript ‘Family Structure and the association with physical activity – findings from 40 Countries participating in the Health Behaviour in School-aged Children (HBSC) ’. We want to thank the reviewers for their constructive and positive feedback about the manuscript, which we believe has improved the quality of our paper. We have addressed all comments and provided a point-by-point response. To clarify, reviewers’ comments are in bold font, and, where appropriate, we have inserted the revised text in blue, with new text highlighted.

Reviewer: 1

**This is a well-written manuscript that needs only a few minor changes**. **There are several places in the paper where you could use the abbreviation for physical activity (PA) instead of the full name.**

Response: We first would like to thank the reviewer for this positive feedback. We have now replaced ‘physical activity’ with the abbreviation throughout the paper. However, we kept the words without abbreviating them in the questionnaire items to reflect the direct wording of the items.

**Figure 2 and Figure 3 are a bit fuzzy and should be adjusted.**

Response: Figures 2 and 3 display the associations between living with one versus two parents and MVPA/VPA across countries. We cannot see in what way these figures are considered unclear. However, to ease the interpretation we have now described the direction of the point estimates the first time we refer to the figures as follows:

Living with one parent was only associated with a lower likelihood of MVPA in Norway, Bulgaria and Iceland (estimate points to the left of the red line), whereas living with one parent was associated with a higher likelihood of MVPA in French Belgium.

**Please state the novelty and practical implications of your study**

Response: We thank the reviewer for this suggestion. We believe that the novelty of the study has been expressed implicitly in several places in the introduction by highlighting the gaps in the literature and stating the purpose of the study. To add to this, we have now more explicitly stated the novelty by rewriting the following in lines 104-108:

From a socio-ecological perspective (11), the current study will add to the existing literature by providing an extensive cross-country examination of the relationship between family structure and PA, which opens up a broader understanding of how environmental factors, policies, and the organisation of adolescent sport, may interplay with an individual’s PA across family structures.

In the discussion section in the conclusion paragraph, we have made the following changes to summarize the study findings and the novelty:

As the structure of families is changing in many countries, studying the associations between family structure and PA is important at this time. Pooled data demonstrated that family structure correlated with daily 60 min MVPA and ≥ 4 times/week VPA. Still, cross-country differences in associations between living with one versus two parents and PA highlight the value of collecting comparable cross-national data on adolescent health behaviours and their social contexts.

For practical implications of the study, we agree with the reviewer that we could have stated it more clearly. In the discussion section, we have therefore added a paragraph explicitly on the practical implications as follows:

Implications

Policymakers, the sports sector, and health professionals should know that young people’s family context can be complex and potentially affect their involvement in PA, especially leisure time VPA. The present findings suggest that existing welfare policy does not necessarily eliminate family structure differences. The role of family support and the structural organisation of youth sports should be addressed in public PA initiatives, and policy actions should support families with limited time and poorer access to PA facilities. Also, as the proportion of children and adolescents living in single-parent or stepparent families continues to grow, it is important to monitor PA habits by family structure, together with overall population trends. Lastly, the current study should also be considered from a broader public health perspective, as unfavourable PA levels add to several other negative health behaviours observed among adolescents living in single-parent or stepparent families, for example, dietary behaviours, smoking, and substance use (22–25).

## Reviewer: 2

**My major concerns are as follows: (1) Not all factors associated with PA were controlled for; for example, caregiver’s BMI and PA could largely affect child’s BMI and PA, which is not accounted for in this paper;** **(2) a family structure could signal other issues in the family, leading to omitted variable bias on the family structure variable;**

Response: We agree with the reviewer that not all potential factors associated with PA are included in the analysis. We did not have data on the caregiver’s PA, which potentially could be associated with the child’s PA. On the other hand, we are not convinced that caregiver’s PA in itself would be an important cause of family structure. As such, this variable may not necessarily be a confounder of the relationship between family structure and PA. More generally, we are limited by the variables that are available in the dataset and by the design of the study, both of which we acknowledge in the limitations paragraph of the discussion.

**(3) the countries in the sample are extremely varied and have very different SES levels and community supports for family and PA (including built environment, sports facilities, school curriculum) – it is possible that fixed effects are too limited to account for these differences;**

Response: The fixed effects model does account for all country-level variation, but it’s true that the fixed effects model does not allow us to examine the extent to which certain country-level factors explain the variation in PA across countries, but that was not the aim of our study. Our second aim, relevant in this context, was to examine cross-country variations in the associations between living with one versus two parents in PA. The fixed effects model does allow us to study this by the inclusion of cross-level interactions. We have added a reference on this in the statistics section:

*Möhring K (2012) The fixed effects approach as alternative to multilevel models for cross-national analyses. In: 10th ESPAnet Conference, Edinburgh, Scotland, 2012.*

**(4) ORs in a model with a non-rare outcome over-inflates the relative risk and should not be used.**

Response: We agree with the reviewer that the interpretation of odds ratio as relative risk can be cumbersome for non-rare outcomes. We have now re-run all analyses using Poission regression, which led to small adjustments to the estimates presented in the previous version of our manuscript. The tables and the text have been updated accordingly throughout the paper. The following revised text was added to the statistics section:

Multilevel Poisson regressionanalysis was used to examine the associations between family structure and measures of MVPA and VPA. Level-1 units were students, and level-2 units were classes. All countries were pooled together for analysis, and the country variable was modelled as a fixed effect (58). We started with a simple random intercept model with family structure as the only covariate (model 1). In the next steps, all level-1 predictors (gender, age, individual-level SES, stepparent in main home, grandparent(s) in main home, siblings in main home, and country) were first added as main effects (model 2), followed by a model that included the 2-way interactions of the model 2 predictors with family structure. Non-significant interactions based on the Wald-test were deleted and the model was re-run with significant interactions only (model 3). To examine whether potential country variations in the association between family structure on the one hand and MVPA and VPA, on the other hand, could be explained by geographical region or country-level family affluence, the country-by-family structure interaction of model 3 was replaced by the two cross-level interactions that included the mentioned country-level variables and family structure (model 4a: geographical region; model 4b: geographical region and country-level family affluence).

As there was a significant interaction between living with one versus two parents and siblings for VPA, the following text was included:

The interaction analysis showed that the strength of the association between living with one versus two parents and VPA was more pronounced for adolescents with siblings than those without siblings (Wald test for siblings interaction: (χ^2^(1)=4.19, *p*=0.04)). The model-derived conditional IRR for those with siblings was statistically significant (IRR=0.93, 95% CI: 0.91, 0.96), whereas the conditional IRR for those without siblings was not (IRR=1.00, 95% CI: 0.94, 1.05).

In the discussion section, the text was revised as follows:

A direct effect of having a sibling(s) in the main house was, not observed for leisure time VPA for at least 4 timers/week. However, the strength of the association between living with one versus two parents and VPA was more pronounced for adolescents with siblings than those without siblings, with a negative significant impact on VPA of having siblings in the main home observed. This finding somewhat nuances the existing literature where a positive relationship has been found between having a sibling and children’s participation in sport (12). It should be mentioned that our outcome measure, > 4 times/week VPA, reflects a relatively high level of PA engagement that may be less feasible for adolescents in one-parent families with siblings, particularly if it demands substantial parental support regarding time and logistics.

Amendments was also done to the results in the abstract as follows:

This impact on MVPA differed across individual-level SES (high SES; IRR=0.92, (p <0.05), low SES; IRR=1.04, (p <0.05)), and was for VPA only significant for those with siblings (IRR=0.93, 95% CI: 0.91, 0.96).

**More detailed comments are below.**

**I wonder if variance decomposition could be used to see how different family structures contribute to the variation in PA (compared to other variables).**

Response: We thank the reviewer for this suggestion, and although this would be interesting in itself, we consider this to be beyond the scope of the present article. We prefer to maintain living with one versus two parents as our primary focus.

**Line 149: If having parent + stepparent was included as a separate binary covariate in the regression, the model assumes that one can have one parent and parent+stepparent at the same time (which is impossible). I wonder if “parent + step parent” should be included as a fourth category in the main covariate (one parent, 2 parents, parent + step parent, no parents). This could also be done in a separate model.**

Response: We thank the reviewer for pointing this out, but this was an unfortunate mistake in writing. The “one parent +” should not have been there and has now been deleted from the text.

**Line 170: Would it be possible to treat MVPA and VPA as a continuous variable as a supplemental analysis? Dichotomizing a variable leads to some loss of information, and ORs often represent an inflated measure of risk.**

Response: The original MVPA and VPA are based on 7 and 8 categories respectively. Although we agree with the reviewer that dichotomizing these variables leads to some loss of information, the results and overall patterns remain the same when treating these variables as continuous. For VPA, the categories are not equally distanced, so we don’t think it would be right to treat this variable as continuous. Number of days per week (MVPA) may not be ideal as a continuous variable either. We have added a line to the results section that treating these two variables as continuous did not change the overall results. We hope this is considered a sufficient solution by the reviewer. Note that we now report risk ratios now and not odds ratios.

**Line 180: Odds ratios (as mentioned above) represent an inflated measure of risk. While logit models are appropriate for use with binary variables, odds ratios can only be used to approximate relative risk when the outcome is rare. In the descriptive statistics, the outcome ranges from 9% to ~30%, making this not a rare outcome. In this case, authors should estimate relative risk instead of using odds ratios. Given that authors used Stata, it is very straightforward to obtain measures of both absolute difference in risk and relative risk from any model, including logit. Please refer to the following publications for methods:
Kleinman, Lawrence C., and Edward C. Norton. "What's the risk? A simple approach for estimating adjusted risk measures from nonlinear models including logistic regression." Health services research 44.1 (2009): 288-302.
Norton, Edward C., Morgen M. Miller, and Lawrence C. Kleinman. "Computing adjusted risk ratios and risk differences in Stata." The Stata Journal 13.3 (2013): 492-509.**

Response: See response to the previous comment about this issue.

**Line 198: I wonder if the authors have attempted to test other ways of controlling for country differences. The sample includes extremely different countries with different supports for families and PA. It would make sense to test for both fixed and random effects, as well as looking at certain regions separately. I would like to see some evidence that a fixed effects model is the best model for this type of sample.**

Response: Country level differences are controlled for by adding country as a fixed effect. Treating country as a random effect would be relevant if our aim was to examine country-level factors that could explain country-level variation in MVPA and VPA, but as mentioned earlier this was not an aim of the study. We did examine to what extent geographical region could explain the variation in the association between family structure and MVPA and VPA.

**Line 195: Why wasn’t the country-level SES used in the models? What about other household characteristics (such as age, gender, BMI, PA of primary caretaker, BMI of the child, etc)?**

Response: We used country-level family affluence (FAS) to capture country-level variation in SES to explain country-level variation in the association between family structure and MVPA/VPA. This aggregated country-level FAS measure is considered a valid SES indicator also for cross-national comparison *(Boyce W, Torsheim T, Currie C, Zambon A. The family affluence scale as a measure of national wealth: validation of an adolescent self-report measure. Social indicators research. 2006;78:473-87.)* Age and gender are controlled for in the analyses. We did not have data on age, gender, BMI and PA of the primary caretaker. This has now been added to the limitation part as follows:

In this study, we lack information on how long the participants lived in their current family structure, the PA motivational climate in the family (16), and other household characteristics (e.g., BMI and PA of the primary caretaker), limiting our ability to study the processes involved.

BMI of the child was not included because we didn’t consider this a likely confounder of the relationship between family structure and MPVA/VPA. For the same reason we did not think PA of the primary caretaker would be. We actually tested BMI as a covariate during the drafting of our manuscript, and this did not alter the results in any way.

**Line 195: It would be helpful to clearly list all models with all of their covariates in each model; otherwise, it is hard to understand what covariates were included and how – from the Statistics paragraph as written. Perhaps include this as a supplemental table if there is no space in the text.**

Response: We added some more details on the model specifications in the statistics paragraph to clarify what covariates were included. The paragraph now reads:

We started with a simple random intercept model with family structure as the only covariate (model 1). In the next steps, all level-1 predictors (gender, age, individual-level SES, stepparent in main home, grandparent(s) in main home, siblings in main home, and country) were first added as main effects (model 2), followed by a model that included the 2-way interactions of the model 2 predictors with family structure. Non-significant interactions based on the Wald-test were deleted and the model was re-run with significant interactions only (model 3). To examine whether potential country variations in the association between family structure on the one hand and MVPA and VPA, on the other hand, could be explained by geographical region or country-level family affluence, the country-by-family structure interaction of model 3 was replaced by the two cross-level interactions that included the mentioned country-level variables and family structure (model 4a: geographical region; model 4b: geographical region and country-level family affluence).
